# Human Adenocarcinoma Cell Line Sensitivity to Essential Oil Phytocomplexes from *Pistacia* Species: a Multivariate Approach †

**DOI:** 10.3390/molecules22081336

**Published:** 2017-08-11

**Authors:** Alessandro Buriani, Stefano Fortinguerra, Vincenzo Sorrenti, Stefano Dall’Acqua, Gabbriella Innocenti, Monica Montopoli, Daniela Gabbia, Maria Carrara

**Affiliations:** 1 Maria Paola Belloni Center for Personalized Medicine, Data Medica Group (Synlab Limited), 35100 Padova, Italy; stefano.fortinguerra@gmail.com (S.F.); vincenzosorrenti88@gmail.com (V.S.); 2 Department of Pharmaceutical and Pharmacological Sciences, University of Padova, 35100 Padova, Italy; stefano.dallacqua@unipd.it (S.D.); gabbriella.innocenti@unipd.it (G.I.); monica.montopoli@unipd.it (M.M.); daniela.gabbia@gmail.com (D.G.); maria.carrara@unipd.it (M.C.); 3 Department of Biomedical Sciences, University of Padova, 35100 Padova, Italy

**Keywords:** *Pistacia*, terpenes, cytotoxic, principal component analysis

## Abstract

Principal component analysis (PCA) multivariate analysis was applied to study the cytotoxic activity of essential oils from various species of the *Pistacia* genus on human tumor cell lines. In particular, the cytotoxic activity of essential oils obtained from *P. lentiscus*, *P. lentiscus* var. chia (mastic gum), *P. terebinthus*, *P. vera*, and *P. integerrima*, was screened on three human adenocarcinoma cell lines: MCF-7 (breast), 2008 (ovarian), and LoVo (colon). The results indicate that all the *Pistacia* phytocomplexes, with the exception of mastic gum oil, induce cytotoxic effects on one or more of the three cell lines. PCA highlighted the presence of different cooperating clusters of bioactive molecules. Cluster variability among species, and even within the same species, could explain some of the differences seen among samples suggesting the presence of both common and species-specific mechanisms. Single molecules from one of the most significant clusters were tested, but only bornyl-acetate presented cytotoxic activity, although at much higher concentrations (IC_50_ = 138.5 µg/mL) than those present in the essential oils, indicating that understanding of the full biological effect requires a holistic vision of the phytocomplexes with all its constituents.

## 1. Introduction

Herbal medicine is emerging as a holistic tool in novel personalized medicine approaches, especially in primary and secondary prevention [1]. In addition to being a primary source of drugs, phytocomplexes, thanks to their multiple active components, can represent effective strategies to integrate pharmacological therapies or, like in Traditional Chinese Medicine, be themselves therapeutic multitarget agents [2,3].

Plants are a major source of chemopreventive phytochemicals [4]. Among these molecules, some terpenes have been shown to exert cytotoxic activity on tumoral cells in different in vitro, in vivo, and clinical studies [5,6,7,8,9]. d-Limonene, one of the most widespread natural monoterpenes, exerts in vitro anti-tumor activity against several cancer types: neuroblastoma, leukemia, breast, skin, liver, lung, and stomach cancer [10,11,12,13,14,15]. Similarly, other dietary monoterpenes, like geraniol, carvacrol, thymol, eugenol, *p*-cymene, 1,4-terpineol, α-terpinene, α-copaene, α-elemene, α-humulene, and α-caryophyllene, among others, have shown significant cytotoxic activity in different cancer cell lines [8,15,16,17,18,19,20,21].

*Pistacia*, a genus of flowering plants from the family Anacardacee, includes several species largely distributed and rich in terpenoids [7,22], which represent a large portion of their essential oils. The *Pistacia* genus comprises more than twenty species and, among them, *P. lentiscus*, *P. terebinthus*, *P. vera*, *P. integerrima*, and *P. lentiscus* L. var. chia are more popular and have been extensively used in traditional medicine for their tonic, antiseptic, antihypertensive, and gastro-protective properties [7,22,23]. More recently, different pharmacological activities, including antioxidant, antimicrobial, antidiabetic, anti-inflammatory, and antitumor activities have been reported [7].

Most published data are related to the resin of *P. lentiscus* L. var. chia (known as mastic gum) which has been reported to exert cytotoxic activity on tumor cells and inhibition of cancer cell proliferation, angiogenesis, and inflammatory response [7,24,25,26,27,28,29,30,31].

A number of studies concerning the cytotoxic activity of other *Pistacia* species have been carried out, mostly using extracts and not terpenes-rich essential oils [7,32,33,34,35,36,37,38]. In some cases, essential oils were shown to possess cytotoxic activity on different tumor cell lines [36,39]. In the present study, we first screened the cytotoxic potential of different essential oils from several species (*P. lentiscus*, *P. lentiscus* L. var. chia, *P. terebinthus*, *P. integerrima*, and *P. vera*) on three human adenocarcinoma cell lines: MCF-7 (breast), 2008 (ovarian), and LoVo (colon). A multivariate analysis approach was then used to investigate the statistical correlations among the different phytocomplex components of the essential oils, allowing the identification of clusters of molecules cooperating in the cytotoxic activity.

## 2. Results and Discussion

### 2.1. Chemical Analysis of Essential Oils

Essential oils were obtained by hydrodistillation of aerial parts of the plants, or obtained from producers, and oil composition was determined using GC-MS. Table 1 and Table 2 show the main components of oil samples from *P. lentiscus* L. (including *P. lentiscus* L., var. chia), *P. terebinthus* L., *P. vera* L., and *P. integerrima* J.L. Stewart ex Brandis, as identified by the Wiley mass spectra library. A total of 32 samples from different aerial parts of the plants were analysed. Although most terpenes were present in both *P. lentiscus* and *P. terebinthus* samples, the oil composition varied considerably, with limonene and *cis*-ocimene being more meaningfully present in samples from *P. terebinthus*, while β-phellandrene, 1,4- and α-terpineol, α- and γ-terpinene, and T and α-cadinol were relatively more abundant in samples from *P. lentiscus*. Collectively, the two species show similar amounts of α- and β-pinene, β-caryophyllene, and butylated hydroxy toluene. Among the samples from other species, those from *P. integerrima* showed similarities to *P. terebinthus* in the content of α- and β-pinene and of β-caryophyllene, and to *P. lentiscus* in the amounts of 1,4- and α-terpineol, endoborneol, and bornyl acetate. Finally, a significant amount of δ-carene appears to be characteristic of all the three species. Oil samples from *P. lentiscus* var. chia (resin) and from *P. vera* (nuts) are the ones with the least similarities with the others. The composition of the essential oils indicates consistency with the major terpenes known to be present in these species [7].

### 2.2. Cytotoxic Activity of Essential Oils on Human Tumor Cell Lines

The cytotoxic activity of the phytocomplexes from the different *Pistacia* species was evaluated in vitro on three human adenocarcinoma cell lines: MCF-7 (breast adenocarcinoma); 2008 (ovarian adenocarcinoma); and LoVo (colon adenocarcinoma). Results are shown in Table 3.

Most oil samples displayed some cytotoxic activity on one or more cell lines with different potencies. In particular, looking at the results obtained with LoVo cells, all the samples from *P. lentiscus* were active, with the exception of the one from *P. lentiscus* var. chia. Samples from *P. terebinthus* displayed a high frequency of activity, above 70%, while both samples from *P. integerrima* were active and the one from *P. vera* was not. The sample with the most activity was one essential oil from *P. lentiscus* leaves (sample A: IC_50_ = 173.9 µg/mL).

Apart from *P. lentiscus* on LoVo cells, none of the species was active on all the other cell lines. In particular the activity towards 2008 cells was higher for the samples from *P. terebinthus* (71%), while *P. lentiscus* displayed a significant activity in 55% of samples, while the two samples from *P. integerrima* were active and those from *P. vera* and *P. lentiscus* var. chia were not. The two most active samples were both from *P. lentiscus* leaves (sample A: IC_50_ = 181.5 µg/mL and sample C: IC_50_ = 220.4 µg/mL).

The activity on MCF-7 cells was higher for the samples from *P. lentiscus* (73%), and it was the only cell line sensitive to the essential oil from *P. vera*. Essential oils from *P. terebinthus* were active in 44% of cases, while the two oils from *P. integerrima* had a weak effect or were inactive. The two most active samples were from *P. lentiscus* leaves (sample B: IC_50_ = 239.6 µg/mL), from *P. terebinthus* galls (sample AB: IC_50_ = 254.9 µg/mL) and from *P. vera* nuts (sample A3: IC_50_ = 290.6 µg/mL).

Taken together the results indicate a dose dependent cytotoxic effect of different *Pistacia* species phytocomplexes on all the three cell lines tested, in most cases with IC_50_ values below 500 µg/mL, suggesting that this effect might be a shared feature of the *Pistacia* genus. There were wide differences in biological activity between samples, regardless of the species and even the anatomical part, likely related to the high variability in oil composition even within the same species, as determined by GC/MS fingerprinting.

The oils did not show a consistent order of potency from one cell line to the other, and in some case the same sample could display high activity on one cell line, while being inactive on the others (Table 3). Such different sensitivity of the three cell lines to the same oils suggests that the cytotoxicity mediating network of molecular targets (from receptors, to second messengers to DNA expression regulators) is not the same in the three cell lines, which then respond in a different fashion to the multiple bioactive compounds present in the phytocomplexes. This aspect is pharmacologically relevant and suggests cell lines’ specific effects.

In general, the biological activity of a phytocomplex is related to its chemical composition, with each single compound acting synergistically with the others. Thus, given the high heterogeneous compositions of essential oils, likely due to genetic diversity of the samples [40], it is difficult to define a unique mechanism of action and indeed, a molecule, or a cluster of molecules, could have an effect on one type of tumor and not on others [9], determining important differences even in essential oils from the same species [41].

Terpenes and terpene-rich essential oils have been shown to exert specific cytotoxic activity on several tumoral cell lines [9,42,43,44,45]. Cytotoxic activity on tumoral cell lines has been previously identified in plants from the *Pistacia* genus, but most studies have focused on different types of extracts and not on the terpene-rich essential oils, suggesting other types of chemicals and mechanisms were involved [7,32,34,38]. In some cases essential oils from *Pistacia* species have been shown to exert cytotoxic activity on tumoral cell lines. *Pistacia vera* and *Pistacia khinjuk* resin essential oils were found to be active against retinoblastoma cells (Y79) [36]. Essential oil from *P. palestina* Boiss. Although inactive on MCF-7 cells, displayed cytotoxic activity towards amelanotic melanoma cells (C32) and renal cell adenocarcinoma (ACHN) [39]. Our results are, thus, consistent with previous observations on the same genus. A significant amount of data has been produced on the cytotoxic, or antiproliferative effect of *P. lentiscus* var. chia, although in most cases not with essential oil [7,30,31]. In some cases essential oil from the resin was used and a significant cytotoxic effect was observed [24,26]. Although we used the same type of essential oil from *P. lentiscus* var. chia resin (mastic), we did not observe any cytotoxic activity in our experimental conditions. This discrepancy could be related to the different experimental models used, to the different treatment with the essential oil, or to the relatively stringent threshold we used for the cytotoxic effectiveness (500 µg/mL). To eliminate nonspecific interferences with adhesion of cells to the experimental substrate, a “pre-incubation” strategy was also used. Unlike the other published data, cell cultures were exposed to essential oils only for three hours and then allowed to recover for the following 21 h. The limitation of unwanted effects on cell adhesion, by improving the focus on cytotoxicity, might explain the different results.

### 2.3. Principal Component Analysis of the Bioactive Essential Oils

**Figure 1:**
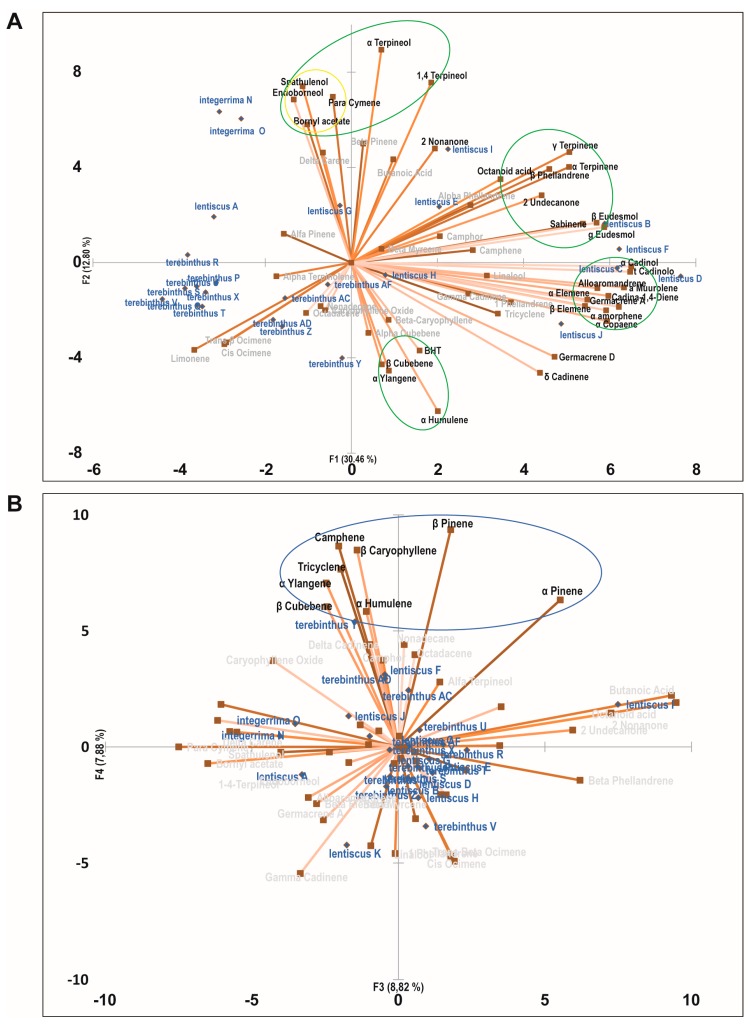
Principal Component Analysis of the cytotoxic effect of *Pistacia* essential oils on LoVo cells. (**A**) PCA biplot with PC1 and PC2 distribution of essential oil samples and chemical components of the phytocomplexes. (**B**) PCA biplot with PC3 and PC4 distribution of essential oil samples and chemical components of the phytocomplexes. Clusters of cooperating compounds with a positive correlation to one or two components are identified with circles (green for *P. lentiscus*, yellow for *P. integerrima*, and blue for *P. terebinthus*).

**Figure 2:**
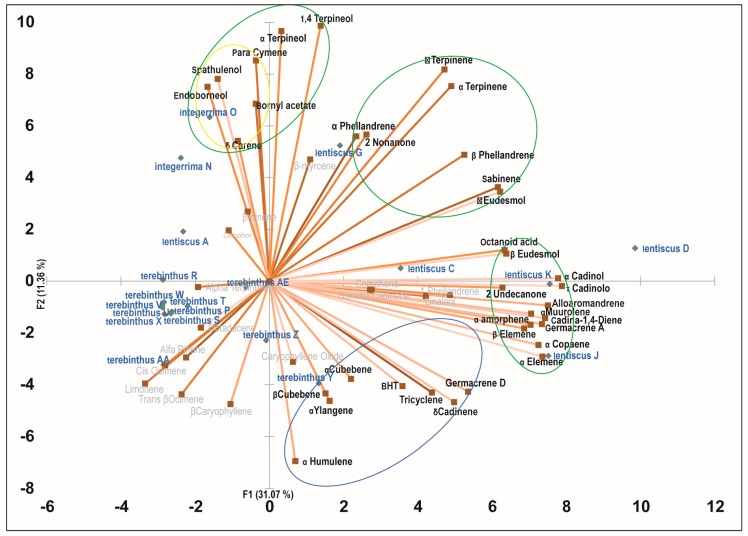
Principal component analysis of the cytotoxic effect of *Pistacia* essential oils on 2008 cells. PCA biplot with PC1 and PC2 distribution of essential oil samples and chemical components of the phytocomplexes. Clusters of cooperating compounds with a positive correlation to one or two components are identified with circles (green for *P. lentiscus*, yellow for *P. integerrima*, and blue for *P. terebinthus*).

**Figure 3:**
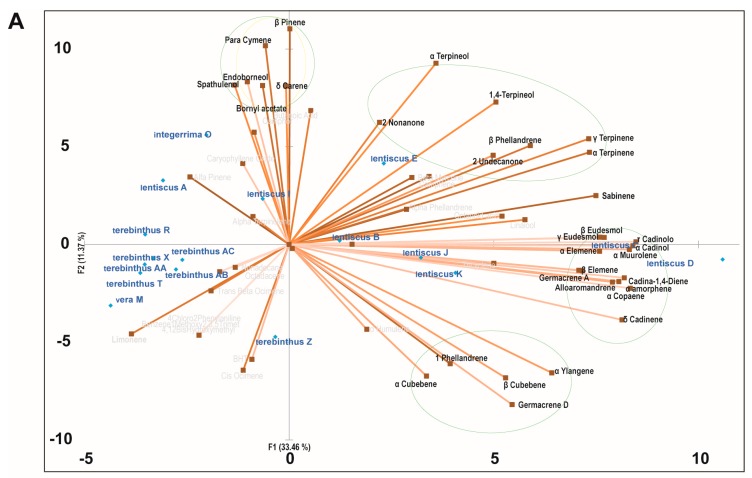

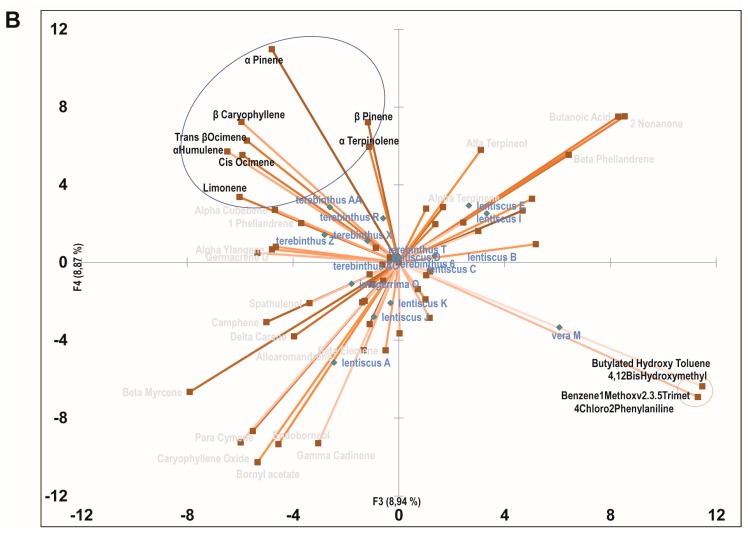
Principal component analysis of the cytotoxic effect of *Pistacia* essential oils on MCF-7 cells. (**A**) PCA biplot with PC1 and PC2 distribution of essential oil samples and chemical components of the phytocomplexes. (**B**) PCA biplot with PC3 and PC4 distribution of essential oil samples and chemical components of the phytocomplexes. Clusters of cooperating compounds with a positive correlation to one or two components are identified with circles (green for *P. lentiscus*, yellow for *P. integerrima*, blue for *P. terebinthus*, and red for *P. vera*).

PCA analysis was performed with the aim of identifying synergic clusters of compounds present in the phytocomplexes, with the best correlation with biological activity (Figure 1, Figure 2 and Figure 3 and Table 4). Four principal components were used (PC1, PC2, PC3, and PC4) which together accounted for more than 60% of the total variance in all three cell lines (Table 4). Positive correlations with one or more principal components were identified for each cell line and each active species in the PC biplot. The highest percent value of association with bioactivity was chosen, proceeding down from the strongest association, until a cluster of active molecules could be identified. PCA allowed the identification of 18 clusters of putative bioactive compounds (compounds likely to cooperate in the cytotoxic activity), many of them differing among cell lines for just one or a few compounds (Table 5). MCF-7 was the cell line with more clusters of bioactive compounds (*n* = 7), while LoVo and 2008 cells had six and five, respectively. All the compounds showing a high level of correlation with bioactivity were included at least in one cluster, with the exception of sabinene and α-humulene, the latter a known anticancer agent [18]. *P. lentiscus* was the species with more clusters of bioactivity-associated molecules in all cell lines. *P. lentiscus* was also the species with higher principal component scores, with L2 being the cluster with the highest values (43.2% LoVo, 42.5% 2008, 44.9 MCF-7).

The active cluster identified for *P. integerrima* on LoVo cells (I1: spathulenol, endoborneol, *p*-cymene, bornyl acetate) is also the core part of I1a and I1b and correlates with activity in all cell lines and, is shared by all L1s *P. lentiscus* clusters, thus suggesting a common mechanism of *P. lentiscus* and *P. integerrima* phytocomplexes throughout the cell lines. Although the aim of the work was not to identify biological activity in single terpenes, given that this particular cluster is shared by all cell lines and at the same time by more than one *Pistacia* species, we tested the cytotoxic activity of the pure compounds on LoVo cells, where the activity of the phytocomplexes seemed to be stronger. Bornyl acetate was the only tested molecule showing a cytotoxic activity when used alone (IC_50_ = 138.5 (130.8 to 146.7 µg/mL), although its potency was not as strong as that corresponding to the actual concentration range in the oils. The other compounds displayed no activity in the pre-treatment paradigm used (data not shown), although *p*-cymene and spathulenol had been previously shown to have anti-tumoral activity [15,42]. This points out to the specific biological effect exerted by the phytocomplex as a whole, which cannot be attributed to a single component but, rather, is likely the result of a complex network of simultaneous biological signals which contribute to the global cytotoxic effect. The other cluster whose core compounds are all shared in all the three cell lines is L3 (from *P. lentiscus*): α-copaene, α-cadinol, τ-cadinol, cadina-1,4-diene, α-elemene, α-amorphene, germacrene A, α-muurolene, β-elemene, and alloaromandrene. In this cluster α-copaene and β-elemene are known cytotoxic compounds for tumoral cells [8,17]. α-Terpinene, α-phellandrene, and α-terpinene are shared by L2s clusters, although they represent only one third. In this group α-terpinene has been previously documented for its cytotoxic activity on cancer cells [15,17]. Other active compounds are associated with bioactivity on all cell lines, but seem not to cooperate consistently with the same clusters. α-Terpineol and 1-4 terpineol cooperate with cluster L1 and L1a on LoVo and 2008 cells, respectively, while on MCF-7 cells, though active, cluster with L2b. Both compounds have been previously found to exert in vitro anti-tumoral activity [15,17]. α-Humulene, another terpene previously shown to be cytotoxic in vitro [18], is present in clusters of activity in all the three cell lines from both *P. lentiscus* and *P. terebinthus* (L4, T1, T2, T3). Ten more compounds are differentially cooperating in clusters of cytotoxic activity towards all cell lines, some in the same group, some in totally different contexts (γ-eudesmol: L2, L2a, L3; sabinene: L2, L2a, S; 2-nonanone: S, L2a, L2b; 2-undecanone: L2, L3a, L2b; β-eudesmol: L2, L3a, L3b; germacrene D L4, T2, L4a; β-cubebene L4, T1, T2, L4a; α-ylangene: L4, T1, T2,L4a; δ-cadinene L4, T2, L3b; butylated hydroxytoluene: L4, T1, T2, V1), the last one being the only clustered molecule shared by *P. vera* with other species. Other compounds’ bioactivity are not shared by all three cell lines, thus suggesting a different cell sensitivity and cell-specific mechanisms. Two such molecules, β-caryophyllene and d-limonene, previously shown to have cytotoxic activity and considered promising potential anti-tumorals [8,9,15,42,45] are active only on MCF-7 cells as part of the T3 cluster.

In summary, PCA allowed the identification of 46 compounds in the phytocomplexes correlated with potential biological activity, most of which had not been previously reported for their in vitro cytotoxic activity on tumoral cells (80%). The compounds are distributed within different clusters of molecules potentially cooperating to achieve the cytotoxic activity on the cell lines.

Taken together, the PCA results indicate that the cytotoxic activity of the phytocomplexes can be highlighted in several clusters of likely-synergizing compounds. *P. lentiscus* is the species with the most clusters, which is in line with its higher potency and frequency of activity with respect to the other species tested. Only two of such clusters are common for all the cell lines, they are characteristic of *P. lentiscus*, and one is also shared with *P. integerrima*. In other cases clusters are shared only for one or two cell lines. In the case of *P. vera*, only one cluster could be identified. These similarities and differences suggest that the cytotoxic effect is complex. Some are common for all three cell lines which suggests a shared mechanism for all three adenocarcynomas, but some are not and *P. lentiscus* seems to display the most variability, while samples from the other species seem more consistent throughout the different cell lines. This behaviour points to different mechanisms exerted in a cell-specific fashion.

Keeping in mind that the results with PCA do not necessarily explain the full activity of the whole phytocomplex, the analysis allowed the identification of substances, mostly terpenes, sesquiterpenes, and fatty acids, present in the essential oils likely playing a central role in the in vitro anti-tumoral effect. Of these, some had been previously shown to have such activity when used as single compounds. Almost all the substances identified cooperate with others, thus forming clusters of activity, often different for one or more components in terms of cell sensitivity.

Finally, although all the compounds identified had a positive correlation with the activity, none of the single clusters or the single components are, by themselves, sufficient to justify the whole activity, and at least all the molecules identified contribute to the biological effects.

## 3. Materials and Methods

### 3.1. Chemicals

Methanol, ethyl acetate, formic acid, acetonitrile, and HPLC-grade solvents were purchase from Carlo Erba Reagents Srl (Dasit Group SpA, Cornaredo, Italy). The solvents were of analytical grade. Double distilled water was used in the HPLC mobile phase. All other reagents, including single purified terpenes, were obtained from Sigma Aldrich srl. (Milan, Italy).

### 3.2. Plant Collection

With the exception of *P. terebinthus* flowers (samples AC, AD, AE) which were collected in Provence (43°57′ N 4°49′ E; Avignon countryside, France), aerial parts (leaves, berries, branches, galls, nuts) of the plants were collected in three different Italian regions: *Pistacia terebinthus* L.: Veneto, Colli Euganei (45°21′50.01″ N 11°40′13.51″ E) (samples P, AB, AF); *Pistacia lentiscus* L.: Tuscany, San Giuliano Terme (43°45′45″ N 10°26′29″ E), Pisa (samples B, C, G, J), Tuscany, Montepescali, Maremma (42°52′58.04″ N/11°05′11.97″ E/42.882788° N) (samples D, E, F, H, I, K); *Pistacia vera* L.: Sicily, Mount Etna (37°45′04.45″ N 14°59′38.49″ E) (sample M). Other essential oils were purchased: *P. lentiscus* essential oil from Portugal (sample A) was obtained from Lifetree Aromatix, John Steel, 3949 Longridge Ave. Sherman Oaks, CA, USA; *P. integerrima* J.L. Stewart ex Brandis essential oil was obtained from Bluebell Herbal Products, Bhaktapur, Nepal (samples N, O); *P. lentiscus* var. chia resin (mastic) essential oil, was obtained from “Anemos” Benetos John Galatoulas George Co, Chios, Karfas RD, Kontari, Chios, Greece (sample L).

Plant parts were recognized by one of the authors (G.I.), University of Padua, Italy. Voucher specimens were deposited at the Department of Pharmaceutical and Pharmacological Sciences of the University of Padova (PL01-11; PT01-17; PV01-1). Plant parts (leaves, berries, branches, flowers, galls and nuts of *P. lentiscus*, *P. terebinthus*, and *P. vera*) were collected, rinsed, dried, and frozen at −21 °C within three hours from collection to preserve their phytochemical composition until the moment of the distillation. The material was then hydrodistilled within a few months. All the samples, before distillation, were minced to obtain the maximum extraction yield and make the process of diffusion of the essence easier [47,48,49,50,51].

### 3.3. Extraction of the Essential Oil

Plant parts were hydrodistilled with a Clevenger apparatus for 4 h. The oils were collected and stored at 4 °C in sealed brown vials until analysis. The mean initial weights of the sample were: leaves (450 g), branches, galls or nuts (250 g), berries (100 g), and flowers (270 g). The yield for the various parts of the plant, express as percent of initial wet weight was: leaves (0.05%), branches (0.06%), nuts (0.02%), berries (0.11%), flowers (0.08%), and galls (0.40%).

### 3.4. Phytochemical Determination of Essential Oil Constituents

The chemical composition of the essential oils obtained from *P. lentiscus*, *P. terebinthus*, *P. vera*, *P. integerrima*, and *P. lentiscus* var. chia was determined by gas chromatography analysis coupled with a mass spectrometer detector (GC/MS), with a Hewlett-Packard 6890-5973 system (Agilent Analytical Instruments Littleton, CO, USA).

The identification of compounds by GC-MS was performed considering not only the matching of mass spectra fragmentation but also the retention index. These two parameters allow the identification of phytoconstituents of the essential oil. In particular, the separation was achieved using a HP-5 MS capillary column (30 m × 0.25 mm i.d. × 0.25 film thickness), with a stationary phase of 95%polydimethylsiloxane. GC-MS analysis carried out under the following conditions: carried gas, He; flow rate, 0.8 mL/min; split ratio, 1:10; injection volume, 1 µL; injector temperature, 200 °C. The temperature programme was as follows: 60 °C with 3 min initial hold, and then to 280 °C at a rate of 3 °C/min, and finally held isothermally for 5 min. The ionization mode was electronic impact at 70 Ev. Identification of the components was achieved by comparison of their mass spectral fragmentation patterns with those stored in the data bank (Wiley Library) [47,48,49,50,51]. The percentage composition of the oil was calculated from GC peak areas using the normalization method without correction factors. The data are reported as mean values of four oil injections.

### 3.5. Sample Preparation for Cell Assays

*Pistacia* oils were dissolved in DMSO and then diluted in culture medium to obtain stock solutions which were stored at −20 °C. Each final stock solution contained 1% DMSO, 9% essential oil, and 90% culture medium. All the procedures were carried out under sterile conditions. Before each experiment the stock solutions were diluted with growth medium and used immediately. The final DMSO concentration was 1%.

### 3.6. Cell Lines

Cytotoxicity was evaluated on three human adenocarcinoma cell lines: ovarian (2008) breast (MCF-7), and colon (LoVo). The cell line 2008 was kindly supplied by Prof. G. Marverti (Department of Biomedical Sciences, University of Modena, Reggio Emilia, Italy) and was maintained in RPMI 1640 medium (Lonza, Basel, Switzerland). MCF-7 cells were supplied by the Experimental Zooprophylactic Institute of Lombardy and Emilia (Brescia, Italy) and were grown in Eagle’s Minimum Essential Medium (Lonza, Basel, Switzerland). LoVo cells were supplied by Dr. G. Toffoli (Oncologic Reference Centre, Aviano, Italy), and were grown in HAM’s F12 (Lonza). L929 fibroblasts (supplied by Lombardy and Emilia Romagna Experimental Zootechnic Institute, Brescia, Italy) and HFFF2 cells, a human Caucasian foetal foreskin fibroblast derived from a 14–18 old human foetus (ICLC cell bank, Genova, Italy), were used as healthy controls to verify the specificity of the cytotoxic effects for cancer cells (data not shown). L929 and HFFF2 cells were grown in RPMI 1640 medium (Lonza, Basel, Switzerland) and in DMEM (Seromed-Biochrom KG, Berlin, Germany) respectively, and 1% penicillin/streptomycin (10,000 U/10,000 μg/mL) (Seromed-Biochrom KG). All cultured media were supplemented with 10% heat-inactivated FCS (foetal calf serum) (20% for HFFF2 cells), 1% antibiotics (100 U/mL penicillin and 100 µg/mL streptomycin) (Seromed-Biochrom KG), and 1% 200 mM glutamine (Merck, Darmstadt, Germany). Cells were maintained under humidified conditions (air 95%; carbon dioxide (CO_2_) 5%, at 37 °C).

### 3.7. Cytotoxicity

Cells (1 × 10^5^ cells/mL) were seeded in 96-well tissue plates (Falcon, New York, NY, USA) and treated for 3 h with different concentrations of *Pistacia* essential oils, with single compounds, or with vehicle (0.1% DMSO, untreated controls). The cells were then rinsed with sterile PBS and incubated in culture medium for 21 h. The cytotoxic effect was evaluated by MTT reduction assay after 21 h of incubation. An amount of 20 µL of MTT solution (5 mg/mL in PBS) was added to each well, and plates were incubated for 4 h at 37 °C. DMSO (150 µL) was added to all wells and mixed thoroughly to dissolve the dark-blue crystals. The absorbance was measured on a micro-culture plate reader (Titertek Multiscan, Midland, ON, Canada) using a test wavelength of 570 nm and a reference wavelength of 630 nm. The 3 h pre-incubation followed by recovery model was chosen after some preliminary tests aimed at identifying the best conditions to assay for specific cytotoxic effects. Cisplatin was used as the positive control. IC_50_ values below 500 µg/mL were taken into consideration for the cytotoxic activity of each phytocomplex tested. This value was chosen as threshold in line with recent literature on the subject and other works on cytotoxicity of *Pistacia* essential oils, although some authors have used different thresholds [30,43,44]. For each assay the experiments were performed at least three times and each essential oil was tested in triplicate on the different cell lines.

### 3.8. Statistical Analysis

The results were statistically evaluated by Student’s *t*-test (two-tailed). IC_50_, dose-response curve best-fit, Pearson correlation, and linear regression analysis were performed using GraphPad Prism Version 3.0. Principal component analysis (PCA) was performed using XLSTAT^®^ software (©Addinsoft SARL, New York, NY, USA). PCA was performed on a correlation matrix (Pearson (n)), computed with the contribution (%) of each compound to the IC_50_ of each oil sample, calculated with the following formula:

$$\left(IC50\;\frac{\mu g}{ml}\right)*\frac{compound\;\%}{100}$$

Correlating the amount of each compound with the relative IC_50_ value takes into consideration the intrinsic contribution of the compound to the cytotoxic effect of the phytocomplex and allows the evaluation of its relative potency in the context of the phytocomplex. The resulting values were used as variables in the matrix, and are the vectors in the biplot graphs of principal components, while the samples of the different *Pistacia* species represent the observations in the matrix and the points in the graph. The clusters of molecules in a given species were identified in the graphs according to four vector characteristics: correlation with the *Pistacia* species, positive distribution with respect to one or more given principal component, distance from the origin, and angle widths between vectors.

## 4. Conclusions

This is the largest study conducted to investigate the in vitro anti-tumoral effect of different species from the genus *Pistacia*. The phytocomplexes in essential oils from four different species of *Pistacia* (*lentiscus*, *terebinthus*, *vera*, and *integerrima*) consistently, though differently, induced cytotoxicity on the three cell lines of human adenocarcinoma, with variable potency and tropism depending on the species, and no significant difference among samples from different aerial parts. In general, this suggests that the in vitro anti-tumoral effect is a shared feature of the *Pistacia* genus, although there are differences that also indicate patterns of activity characteristic for each species and for each adenocarcinoma.

PCA was used to investigate the correlations between the molecular components of the phytocomplexes, most of them terpenes, sesquiterpenes, and fatty acids, and the observed biological activity. This approach appears to be favourable because it takes into consideration the relative contribution of each compound of the phytocomplex to the final effect on the tumoral cells creating a correlation with the measured IC_50_ of the considered essential oils. Merging the chemical composition data and the results of the biological assay by a multivariate approach offers a new approach in the evaluation of the bioactivity of these complex mixture. This approach allows the identification of clusters of compounds that, when present together in the mixture, are correlated with increased bioactivity.

Molecular cooperation is the most important aspect of the biological activity found in natural products, where a holistic approach is needed to understand their therapeutic potential. This was confirmed in our PCA study and we found several clusters of bioactivity-associated molecules in each species, likely to specifically synergize on each cell line. Two such clusters were shared for the three adenocarcinomas, indicating the presence of at least two common pathways of cytotoxic activity, while all the others were different, thus suggesting specific cell line sensitivity and different species-specific mechanisms observed in vitro. Among the molecules identified only about 20% were compounds already known for their anti-tumoral effect as single drugs, while the others were novel findings. Among the new molecules identified, mostly terpenes, bornyl acetate showed significant cytotoxic activity even when used alone, but to a much lesser extent if compared to the dose range present in the essential oil, thus highlighting the importance of cooperation between different molecules in the phytocomplex to achieve the final effect.

## Figures and Tables

**Table 1:** Oil composition of *P*. *lentiscus*, *P*. *lentiscus* var. chia, *P*. *vera*, and *P*. *integerrima*.

RT	Oil Composition	*P. lentiscus*											var. *chia*	*P*. *vera*	*P*. *integerrima*	
		A (l)	B (l)	C (l)	D (l)	E (l)	F (l)	G (be)	H (be)	I (be)	J (br)	K (br)	L (r)	M (n)	N (g)	O (g)
6.08	Tricyclene	-	0.33	0.58	0.14	0.06	1.20	-	-	-	0.90	-	-	-	-	-
6.22	α-Phellandrene	-	1.51	0.12	0.14	0.20	0.15	0.61	-	0.20	-	0.14	-	-	-	-
6.48	α-Pinene	12.95	11.45	12.97	9.17	24.68	12.86	20.13	6.11	40.68	18.15	13.73	72.93	0.99	14.41	22.37
6.94	Camphene	2.05	1.46	2.49	0.52	0.58	4.96	1.06	-	0.98	3.90	0.22	0.58	-	0.94	1.32
7.78	Sabinene	1.16	4.31	4.56	2.87	2.32	4.47	0.99	1.21	0.86	2.83	6.77	0.30	-	2.51	2.46
7.88	β-Pinene	4.33	3.07	4.38	1.15	8.64	4.25	4.13	0.48	7.60	4.19	0.94	2.58	-	5.62	7.63
8.40	β-Myrcene	8.42	0.97	0.67	4.45	0.91	9.23	23.80	57.79	1.08	4.08	5.47	13.57	-	2.81	3.20
8.86	1-Phellandrene	0.69	4.64	0.80	4.60	2.12	2.07	3.84	3.79	0.92	0.81	8.35	-	-	0.56	0.77
9.02	Methylanisol	-	-	-	-	-	-	-	-	-	-	-	0.58	-	-	-
9.07	δ-Carene	0.67	-	-	-	-	-	-	-	-	3.14	-	-	-	4.06	8.36
9.33	α-Terpinene	0.56	4.66	3.60	3.76	3.11	2.82	2.71	0.90	1.13	0.72	1.73	-	-	0.80	1.80
9.69	*para*-Cymene	6.43	0.64	0.22	0.50	0.35	0.30	0.48	0.34	0.16	0.42	0.98	-	-	2.09	3.72
9.81	β-Phellandrene	-	6.48	4.93	5.79	11.35	4.73	6.48	3.44	8.03	-	6.61	-	-	-	-
9.89	Limonene	17.55	-	-	-	-	-	-	-	-	-	-	0.89	1.54	5.94	7.81
10.21	*cis*-Ocimene	-	0.60	0.35	1.00	0.72	0.52	-	0.29	0.22	1.08	0.61	-	-	-	-
10.62	Trans-β-Ocimene	0.14	0.41	0.23	0.53	0.46	0.35	-	0.57	-	0.32	0.23	-	-	-	-
10.96	Butanoic Acid	-	-	-	-	0.55	-	-	-	1.07	-	-	-	-	-	-
11.03	γ-Terpinene	1.24	7.42	5.90	6.11	4.89	4.94	4.93	1.85	2.24	1.51	2.96	-	-	1.52	3.74
12.26	α-Terpinolene	0.61	2.48	2.08	2.35	2.59	1.75	2.08	0.63	2.27	0.64	1.04	-	-	0.57	1.61
12.49	2-Nonanone	-	0.34	0.17	0.19	0.63	0.30	0.44	0.31	1.65	-	-	-	-	-	-
12.60	α-Pinene Oxide	-	-	-	-	-	-	-	-	-	-	-	0.56	-	-	-
12.74	Linalool	0.11	-	0.15	0.22	0.39	-	-	-	-	-	0.36	0.73	-	-	-
12.96	Pinocarveol	-	-	-	-	-	-	-	-	-	-	-	0.21	-	-	-
14.64	Pinocarvone	-	-	-	-	-	-	-	-	-	-	-	0.10	-	-	-
14.70	Camphor	0.13	-	-	-	0.08	0.31	-	-	-	-	-	-	-	-	-
15.74	Endoborneol	6.77	0.12	0.34	-	0.24	0.43	0.32	-	0.65	-	-	-	-	3.12	1.02
16.20	1-4 Terpineol	3.37	14.89	12.26	13.11	7.12	10.43	8.78	4.09	2.63	3.03	6.98	-	2.13	29.52	20.69
16.79	α-Terpineol	0.78	4.92	5.36	6.42	7.41	4.96	7.97	1.11	9.83	1.41	1.13	-	2.56	14.08	8.74
17.48	Myrtenol	-	-	-	-	-	-	-	-	-	-	-	0.18	-	-	-
19.44	Octanoid Acid	-	0.18	0.13	0.22	-	0.19	-	-	0.54	-	0.17	-	-	-	-
21.04	Bornyl Acetate	24.48	2.12	1.77	0.17	-	0.52	1.94	-	-	0.98	0.28	-	-	4.55	1.55
21.39	Verbenone	-	-	-	-	-	-	-	-	-	-	-	0.26	-	-	-
21.42	2-Undecanone	0.30	0.69	0.19	0.55	0.99	0.67	0.41	0.59	1.38	1.47	0.65	-	-	-	-
24.77	α-Ylangene	-	0.42	0.87	0.70	-	0.30	-	0.32	-	0.46	0.60	-	-	-	-
25.39	β-Cubebene	-	-	0.25	0.21	-	-	-	-	-	-	0.34	-	-	-	-
25.47	β-Elemene	-	0.15	0.19	0.23	0.11	0.16	-	-	-	0.52	0.51	-	-	-	-
26.61	β-Caryophyllene	2.09	2.79	3.21	3.24	4.02	4.92	0.44	0.81	1.18	3.91	1.70	0.30	0.60	2.81	1.96
27.85	α-Cubebene	-	-	0.05	0.24	-	-	-	-	-	-	-	-	-	-	-
27.96	α-Humulene	0.17	1.02	0.88	1.13	0.48	1.09	0.25	0.37	-	1.10	0.60	-	-	-	-
28.25	Alloaromandrene	0.10	0.30	0.34	0.39	-	0.24	-	-	-	0.44	0.48	-	-	-	-
28.99	α-Amorphene	0.37	0.85	1.51	1.30	0.17	0.64	0.35	0.80	0.39	1.16	1.13	-	-	-	-
29.10	Germacrene D	0.25	4.71	6.82	5.00	1.37	4.34	2.06	2.76	1.23	8.36	8.32	-	0.62	-	-
29.66	α-Elemene	-	0.33	0.41	0.51	0.26	0.34	-	-	0.35	0.90	0.99	-	-	-	-
29.89	α-Muurolene	0.13	0.71	1.21	1.07	0.33	0.68	-	0.68	0.44	0.98	0.93	-	-	-	-
30.05	Germacrene A	-	0.14	0.18	0.30	0.16	0.23	-	-	-	0.44	0.68	-	-	-	-
30.42	Butylated Hydroxy Toluene	-	0.60	0.81	1.36	0.44	1.14	0.88	1.23	1.72	4.33	1.25	-	15.74	-	-
30.81	δ-Cadinene	0.20	2.82	4.80	4.40	1.11	2.52	1.32	3.28	1.74	4.44	3.99	-	0.66	-	-
32.26	Spathulenol	-	-	-	-	-	-	-	-	-	-	-	-	-	1.26	0.53
32.78	γ-Cadinene	0.54	-	-	-	-	-	-	-	-	0.19	0.87	-	-	-	-
33.20	Caryophyllene Oxide	2.53	-	-	-	0.12	-	-	-	-	0.83	-	-	-	-	-
34.79	Cadina-1,4-Diene	-	0.83	1.26	1.50	0.20	0.61	-	0.46	-	0.86	0.71	-	-	-	-
34.92	γ-Eudesmol	-	1.21	0.96	1.07	0.48	0.77	0.41	0.63	0.28	-	0.61	-	-	-	-
35.31	α-Cadinol	-	2.50	3.29	4.13	1.63	2.52	0.76	1.96	0.97	3.18	2.70	-	-	-	-
35.45	α-Copaene	-	0.75	1.03	1.29	0.49	0.76	-	0.53	0.31	1.22	0.81	-	-	-	-
35.58	β-Eudesmol	-	0.47	0.31	0.41	0.19	0.28	-	-	0.20	-	0.46	-	-	-	-
35.80	T-Cadinolo	0.85	3.16	3.90	4.73	2.61	3.55	1.15	2.79	1.68	4.53	3.77	-	-	-	-
56.28	Nonadecane	-	-	-	-	-	-	-	-	-	-	-	-	-	-	-
61.56	Octadecane	-	-	-	-	-	-	-	-	-	-	-	-	-	-	-
67.79	CPA ^1^	-	-	-	-	-	-	-	-	-	-	-	-	32.88	-	-
67.96	Benzene1Methox ^2^	-	-	-	-	-	-	-	-	-	-	-	-	25.63	-	-
69.78	4,12BisHydroxy ^3^	-	-	-	-	-	-	-	-	-	-	-	-	4.29	-	-

Different Essential Oil samples were identified progressively using letters from A–O. (l) leaves, (be) berries, (br) branches, (f) flowers, (r) resin, (n) nuts, (g) galls. RT: retention time. Values are percent of the total oil composition. ^1^ 4-chloro-2-phenylaniline. ^2^ Benzene-methoxy-trimethyl-tetrahydro-halenedica-urs-12-en-28-al; ^3^ 4,12-bis(hydroxymethyl)-2,2-metac-4,14-bis(hydroxymethyl)-2,2-metac-1-oxo-2,3,5,6,7,8-hexahydrothienol.

**Table 2:** Oil composition of *P*. *terebinthus*.

RT	Oil Composition	P (l)	Q (l)	R (l)	S (l)	T (l)	U (l)	V (l)	W (l)	X (l)	Y (be)	Z (be)	AA (g)	AB (g)	AC (f)	AD (f)	AE (f)	AF (br)
6.08	Tricyclene	-	-	-	-	-	0.25	-	-	-	0.30	-	-	-	0.48	0.45	-	0.18
6.22	α-Phellandrene	-	-	-	-	-	-	-	-	-	-	-	-	-	0.21	-	-	0.36
6.48	α-Pinene	35.6	12.17	41.03	11.61	14.1	60.9	10.0	10.99	18.7	31.63	8.69	54.19	42.16	28.90	26.43	11.74	28.72
6.94	Camphene	0.55	0.26	1.02	-	-	1.56	0.24	0.37	0.46	1.50	0.48	0.69	0.67	2.12	1.83	-	1.06
7.78	Sabinene	-	0.62	-	0.39	-	0.22	0.15	0.28	-	1.20	0.85	0.65	0.18	2.00	0.91	1.41	4.53
7.88	β-Pinene	1.12	1.14	6.17	1.39	0.71	1.87	0.32	1.48	3.27	7.44	1.39	3.68	1.81	6.40	5.85	2.12	4.07
8.40	β-Myrcene	1.57	1.68	1.37	1.82	2.03	1.98	1.41	1.60	2.90	1.21	1.11	1.05	1.42	1.68	0.97	31.56	1.67
8.86	1-Phellandrene	0.62	0.35	0.49	0.75	0.62	0.47	0.19	0.29	0.27	3.34	7.66	0.49	6.61	0.32	0.37	0.61	0.59
9.02	Methylanisol	-	-	-	-	-	-	-	-	-	-	-	-	-	-	-	-	-
9.07	δ-Carene	-	-	-	1.23	-	0.76	-	-	-	-	-	-	-	-	-	-	-
9.33	α-Terpinene	-	0.43	0.83	0.71	-	0.44	-	0.21	0.34	0.25	0.34	-	-	0.65	0.51	-	1.46
9.69	Para Cymene	-	0.11	-	-	-	0.17	-	-	-	0.38	-	-	0.18	0.20	-	0.79	0.49
9.81	β-Phellandrene	-	-	-	-	-	-	-	-	-	-	-	-	-	-	-	-	-
9.89	Limonene	27.4	50.70	6.09	21.04	60.4	6.16	1.23	8.03	60.2	12.59	32.85	13.97	31.07	28.76	23.03	5.58	6.56
10.21	Cis-Ocimene	19.6	15.64	10.93	20.97	3.86	6.31	63.6	54.19	0.96	6.79	17.99	1.89	0.37	-	3.88	-	13.44
10.62	Transβ-Ocimene	5.49	4.45	3.80	6.04	1.17	1.86	18.3	16.46	0.38	1.66	5.01	0.40	-	-	1.22	-	3.33
10.96	Butanoic Acid	-	-	-	-	-	-	-	-	-	-	-	-	-	-	-	-	-
11.03	γ-Terpinene	0.22	0.69	0.48	0.62	-	0.49	0.17	0.34	0.60	0.33	0.42	0.12	-	1.08	0.77	0.46	2.46
12.26	α-Terpinolene	0.92	0.93	17.07	16.59	4.90	7.13	0.38	0.46	1.76	0.44	1.27	0.26	0.28	0.57	2.99	-	1.08
12.49	2-Nonanone	-	-	-	-	-	-	-	-	-	-	-	-	-	-	-	-	-
12.60	α-Pinene Oxide	-	-	-	-	-	-	-	-	-	-	-	-	-	-	-	-	-
12.80	Linalool	-	-	-	-	-	0.18	-	-	-	-	0.17	-	-	-	-	-	-
12.96	Pinocarveol	-	-	-	-	-	-	-	-	-	-	-	-	-	-	-	-	-
14.64	Pinocarvone	-	-	-	-	-	-	-	-	-	-	-	-	-	-	-	-	-
14.70	Camphor	-	-	-	-	-	-	-	-	-	-	-	-	-	-	-	-	-
15.74	Endoborneol	-	-	-	-	-	-	-	-	-	-	-	-	-	-	-	-	-
16.20	1-4 Terpineol	0.31	1.63	0.29	1.86	1.21	0.29	0.35	0.50	0.46	1.00	0.87	0.18	0.13	2.86	1.55	-	8.91
16.79	α-Terpineol	1.63	4.05	5.22	1.92	1.18	2.23	1.35	1.22	2.87	2.43	0.93	0.35	1.36	1.69	1.45	-	2.26
17.48	Myrtenol	-	-	-	-	-	-	-	-	-	-	-	-	-	-	-	-	-
19.44	Octanoid Acid	-	-	-	-	-	-	-	-	-	-	-	-	-	-	-	-	-
21.04	Bornyl Acetate	-	-	0.47	-	-	-	-	-	-	0.64	0.35	-	0.18	-	-	-	0.23
21.39	Verbenone	-	-	-	-	-	-	-	-	-	-	-	-	-	-	-	-	-
21.42	2-Undecanone	-	-	-	-	-	-	-	-	-	-	-	-	-	-	-	-	-
24.77	α-Ylangene	0.23	0.16	-	-	-	-	-	-	-	4.51	0.96	0.15	0.19	-	2.14	-	0.51
25.39	β-Cubebene	-	-	-	-	-	-	-	-	-	1.60	0.40	-	-	-	0.37	-	0.19
25.47	β-Elemene	-	-	-	-	-	-	-	-	-	-	-	-	-	-	-	-	-
26.61	β-Caryophyllene	3.23	1.38	2.75	4.72	2.00	3.86	0.62	1.29	3.43	4.77	1.64	14.33	0.94	0.97	8.05	0.63	1.06
27.85	α-Cubebene	-	-	-	-	-	-	-	-	-	0.26	0.42	-	-	-	-	-	0.74
27.96	α-Humulene	0.47	0.33	-	0.86	1.07	1.21	-	-	1.46	1.47	0.55	2.15	0.33	0.65	1.24	-	0.22
28.25	Alloaromandrene	-	-	-	-	-	-	-	-	-	-	-	-	-	-	-	-	-
28.99	α-Amorphene	-	0.24	-	-	-	-	0.17	-	0.30	0.55	0.24	-	0.15	0.43	0.48	1.27	0.77
29.10	Germacrene D	0.23	0.62	-	1.56	1.40	-	0.14	0.45	-	2.24	9.42	2.91	7.08	5.87	2.44	4.45	2.91
29.66	α-Elemene	-	-	-	-	-	-	-	-	-	0.18	-	-	-	-	-	-	0.46
29.89	α-Muurolene	-	-	-	-	-	-	-	-	-	0.25	-	-	-	0.33	0.27	0.76	0.47
30.05	Germacrene A	-	-	-	-	-	-	-	-	-	-	-	-	0.14	-	-	-	-
30.42	Butylated Hydroxy Toluene	0.32	0.77	0.37	3.34	2.36	0.62	0.46	0.45	0.55	0.58	0.46	0.30	0.45	1.56	1.58	-	1.82
30.81	δ-Cadinene	0.44	0.67	-	1.32	0.92	0.31	0.31	0.63	0.61	6.42	2.33	0.31	0.55	1.22	3.61	3.06	3.51
32.26	Spathulenol	-	-	-	-	-	-	-	-	-	-	-	-	-	-	-	-	-
32.78	γ-Cadinene	-	-	-	-	-	-	-	-	-	-	-	-	-	-	-	0.84	-
33.20	Caryophyllene Oxide	-	0.23	-	-	-	0.27	-	-	-	0.40	-	0.39	-	-	0.75	-	-
34.79	Cadina-1,4-Diene	-	-	-	-	-	-	-	-	-	0.31	0.17	-	-	-	0.36	-	0.31
34.92	γ-Eudesmol	-	-	-	-	-	-	-	-	-	-	-	-	0.70	-	-	-	-
35.31	α-Cadinol	-	-	-	-	-	-	-	-	-	0.26	-	-	0.21	0.81	0.73	-	0.53
35.45	α-Copaene	-	-	-	-	-	-	-	-	-	0.39	0.55	-	-	0.25	0.19	-	-
35.58	β-Eudesmol	-	-	-	-	-	-	-	-	-	-	-	-	0.21	-	-	-	-
35.80	T-Cadinolo	-	0.17	-	-	-	-	-	-	-	0.31	0.65	-	0.30	1.14	0.87	1.26	0.36
56.28	Nonadecane	-	-	-	-	-	-	-	-	-	-	-	-	-	2.20	1.04	-	-
61.56	Octadecane	-	-	-	0.47	-	-	-	-	0.25	-	-	-	-	1.99	0.71	-	-
67.79	CPA ^1^	-	-	-	-	-	-	-	-	-	-	-	-	-	-	-	-	-
67.96	Benzene1Methox ^2^	-	-	-	-	-	-	-	-	-	-	-	-	-	-	-	-	-
69.78	4,12BisHydroxy ^3^	-	-	-	-	-	-	-	-	-	-	-	-	-	-	-	-	-

Different Essential Oil samples were identified progressively using letters from P–Z and AA–AF. (l) leaves, (be) berries, (br) branches, (f) flowers, (r) resin, (g) galls. RT: retention time. Values are percent of the total oil composition. ^1^ 4-chloro-2-phenylaniline. ^2^ Benzene-methoxy-trimethyl-tetrahydro-halenedica-urs-12-en-28-al; ^3^ 4,12-bis(hydroxymethyl)-2,2-metac-4,14-bis(hydroxymethyl)-2,2-metac-1-oxo-2,3,5,6,7,8-hexahydrothienol.

**Table 3:** Cytotoxic effect of *Pistacia* essential oils.

Samples	LoVo Cells	2008 Cells	MCF-7 Cells
A	173.9 (151.0–200.3) µg/mL	181.5 (166.5–197.7) µg/mL	249.4 (243.5–255.4) µg/mL
	*n* = 3	*n* = 7	*n* = 3
B	410.8 (302.4–558.1) µg/mL	>600 µg/mL	239.6 (189.3–303.3) µg/mL
	*n* = 2	*n* = 2	*n* = 2
C	399.3 (349.6–456.1) µg/mL	220.4 (107.0–454.2) µg/mL	543.1 (503.6–585.7) µg/mL
	*n* = 2	*n* = 3	*n* = 1
D	420.0 (335.7–525.6) µg/mL	395.0 (310.8–502.1) µg/mL	591.4 (272.7–1283) µg/mL
	*n* = 5	*n* = 4	*n* = 2
E	401.3 (323.5–497.8) µg/mL	>600 µg/mL	499.4 (447.9–556.9) µg/mL
	*n* = 4	*n* = 5	*n* = 2
F	461.6 (273.1–780.2) µg/mL	N.A.	>600 µg/mL
	*n* = 3	*n* = 2	*n* = 1
G	438.3 (309.0–621.6) µg/mL	515.9 (435.1–611.6) µg/mL	N.A.
	*n* = 2	*n* = 3	*n* = 2
H	414.4 (362.9–473.3) µg/mL	N.A.	N.A.
	*n* = 2	*n* = 2	*n* = 3
I	539.3 (475.0–612.3) µg/mL	N.A.	299.9 (260.4–345.4) µg/mL
	*n* = 3	*n* = 1	*n* = 2
J	369.1 (334.1–407.7) µg/mL	388.0 (334.5–450.1) µg/mL	356.1 (295.1–429.7) µg/mL
	*n* = 2	*n* = 2	*n* = 3
K	417.7 (392.1–445.1) µg/mL	312.1 (140.6–692.7) µg/mL	311.6 (260.4–373.0) µg/mL
	*n* = 2	*n* = 2	*n* = 2
L	N.A.	N.A.	N.A.
	*n* = 3	*n* = 3	*n* = 3
M	N.A.	N.A.	290.6 (210.2–401.8) µg/mL
	*n* = 2	*n* = 1	*n* = 3
N	444.7 (418.3–472.8) µg/mL	359.0 (328.1–392.9) µg/mL	N.A.
	*n* = 3	*n* = 2	*n* = 3
O	511.2 (427.6–611.2) µg/mL	556.1 (512.2–603.7) µg/mL	515.0 (407.2–651.5) µg/mL
	*n* = 3	*n* = 2	*n* = 2
P	403.1 (255.9–635.0) µg/mL	401.5 (334.7–481.5) µg/mL	N.A.
	*n* = 2	*n* = 3	*n* = 4
Q	473.4 (418.8–535.1) µg/mL	N.A.	N.A.
	*n* = 2	*n* = 1	*n* = 1
R	464.4 (421.9–511.3) µg/mL	464.1 (384.0–560.9) µg/mL	501.2 (429.6–584.7) µg/mL
	*n* = 2	*n* = 2	*n* = 2
S	427.6 (411.7–444.2) µg/mL	337.6 (243.8–467.4) µg/mL	N.A.
	*n* = 2	*n* = 1	*n* = 3
T	425.9 (395.2–458.9) µg/mL	325.0 (238.9–442.1) µg/mL	392.0 (357.1–430.4) µg/mL
	*n* = 2	*n* = 2	*n* = 3
U	406.7 (367.2–450.4) µg/mL	348.1 (263.7–459.5) µg/mL	N.A.
	*n* = 3	*n* = 2	*n* = 3
V	400.4 (270.0–593.6) µg/mL	398.0 (367.0–431.6) µg/mL	N.A.
	*n* = 2	*n* = 2	*n* = 3
W	N.A.	371.6 (291.9–472.9) µg/mL	N.A.
	*n* = 2	*n* = 2	*n* = 3
X	392.1 (310.7–494.8) µg/mL	484.0 (387.2–604.9) µg/mL	572.6 (424.8–771.8) µg/mL
	*n* = 2	*n* = 3	*n* = 2
Y	509.8 (377.6–688.3) µg/mL	598.6 (557.5–642.8) µg/mL	N.A.
	*n* = 2	*n* = 6	*n* = 4
Z	411.8 (276.4–613.6) µg/mL	453.2 (403.7–508.7) µg/mL	512.9 (323.6–812.9) µg/mL
	*n* = 2	*n* = 2	*n* = 2
AA	>600 µg/mL	591.9 (509.2–688.0) µg/mL	503.3 (470.9–537.9) µg/mL
	*n* = 2	*n* = 3	*n* = 1
AB	N.A.	N.A.	254.9 (243.3–267.1) µg/mL
	*n* = 2	*n* = 1	*n* = 2
AC	441.0 (404.4–481.0) µg/mL	N.A.	297.7 (277.6–319.2) µg/mL
	*n* = 3	*n* = 2	*n* = 3
AD	345.2 (297.8–400.1) µg/mL	N.A.	N.A.
	*n* = 2	*n* = 1	*n* = 1
AE	>600 µg/mL	331.4 (266.9–411.5) µg/mL	>600 µg/mL
	*n* = 1	*n* = 1	*n* = 1
AF	405.6 (355.5–462.7) µg/mL	N.A.	N.A.
	*n* = 4	*n* = 1	*n* = 1
CisPlatin	10.7 (7.1–17.9) µg/mL	4.1 (2.6–7.2) µg/mL	45.0 (25.9–71.7) µg/mL
	*n* = 3	*n* = 3	*n* = 3

Different Essential Oil samples were identified progressively using letters from A–Z and AA–AF. Values are means (confidence interval). N.A. (not active).

**Table 4:** Principal component values.

Principal Component	LoVo	2008	MCF7
PC1	30.4%	31.1%	33.5%
PC2	12.8%	11.4%	11.4%
PC3	8.8%	10.6%	8.9%
PC4	8.0%	8.9%	8.9%
Total per cell line	60.0%	62.0%	62.7%

Values are percent accountability of the total variance.

**Table 5:** Cluster comparisons.

Compound	LoVo							2008					MCF7								Reported Cytotoxic Activity
	I1	L1	L2	L3	L4	T1	S	I1a	L1a	L2a	L3a	T2	I1b	L1b	L2b	L3b	L4a	T3	V1	S	
spathulenol																					[42]
endoborneol																					
*p*-cymene																					[15]
bornyl acetate																					
δ-carene																					
α-pinene																					
β-pinene																					
α-terpineol																					[17]
1-4 terpineol																					[15,46]
cterpinene																					
γ-terpinene																					[15,17]
β-phellandrene																					
γ-eudesmol																					
sabinene																					
2-nonanone																					
α-phellandrene																					
2-undecanone																					
β-eudesmol																					
α-copaene																					[17]
α-cadinol																					
T-cadinol																					
cadina-1,4-diene																					
β-elemene																					[8]
α-amorphene																					
germacrene A																					
α-muurolene																					
α-elemene																					
alloaromandrene																					
Octanoid acid																					
germacrene D																					
α-cubebene																					
β-cubebene																					
α-ylangene																					
1-phellandrene																					
BHT ^1^																					
α-cadinene																					
tricyclene																					
α-humulene																					[18]
trans β-ocimene																					
cis ocimene																					
d-limonene																					[8,15]
β-caryophyllene																					[8]
BMTTHU ^2^																					
HMHMOH ^3^																					
CPA ^4^																					
camphene																					

I (integerrima); L (lentiscus); T (terebinthus); V (vera); S = single, unclustered bioactivity-correlated compounds. ^1^ Butylated hydroxy toluene; ^2^ Benzene-methoxy-trimethyl-tetrahydro-halenedica-urs-12-en-28-al; ^3^ 4,12-bis(hydroxymethyl)-2,2-metac-4,14-bis(hydroxymethyl)-2,2-metac-1-oxo-2,3,5,6,7,8-hexahydrothienol; ^4^ 4-chloro-2-phenylaniline.
